# Vesicovaginal reflux: A case report

**DOI:** 10.4103/0971-3026.54882

**Published:** 2009-08

**Authors:** Monali Warade, Yameen Majid, L Dayananda, Kanchan Gupta

**Affiliations:** Department of Radiodiagnosis, Narayana Hrudayalaya Institute of Medical Sciences, 258/A, Bommasandra Industrial Area, Bangalore-560 099, India

**Keywords:** Hydrocolpos, incontinence, micturating cystourethrogram, vesicovaginal reflux, wide bladder neck

## Abstract

Vesicovaginal reflux is a common cause of urinary incontinence in girls. A micturating cystourethrogram, which is the diagnostic investigation of choice, can demonstrate retrograde filling of the vagina during micturition and the complete emptying of the vagina at the end of micturition. Vesicovaginal reflux is a rare cause of gross hydrocolpos occurring without any anatomical obstruction. The condition may be associated with functional voiding disturbances.

## Introduction

Hydrocolpos is commonly due to an anatomical obstruction such as imperforate hymen, vaginal septum or hypoplastic vagina. Gross distention of the vagina due to refluxed urine has been rarely described. Vesicovaginal reflux (VVR) is a common entity known to be associated with physiologic and pathologic incontinence in the pediatric age group. We report one such case of VVR with classical manifestations.

## Case Report

**Figure 1(A–D):**
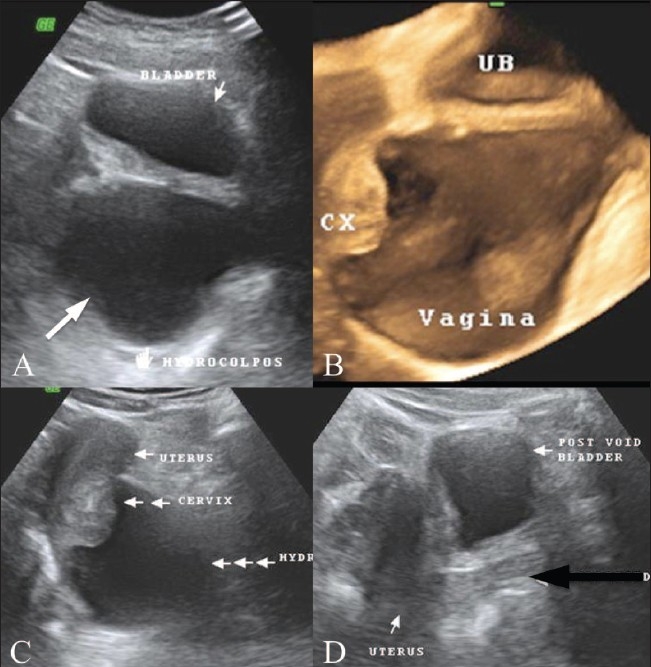
USG pelvis (A) shows a grossly distended fluidfilled vagina (arrow), posterior to the urinary bladder suggestive of hydrocolpos. 3D reformatted image (B) demonstrates a grossly distended, fluid-filled vagina, suggestive of hydrocolpos; the cervix (CX) is suspended at its upper end, posterior to the urinary bladder (UB). USG pelvis (C) shows a normal uterus and cervix (note the distended vagina – three horizontal arrows). Postmicturition USG (D) shows complete evacuation of the vaginal fluid (black arrow) and a urinary bladder post-void residue

**Figure 2(A–B):**
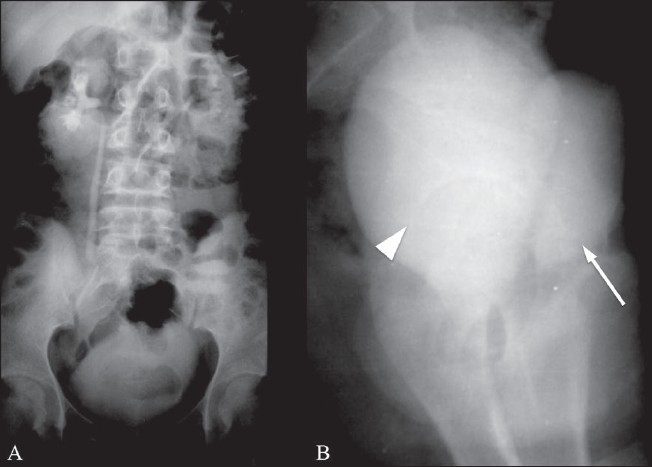
Intravenous urogram. AP projection (A) shows normal renal collecting systems without ureteric ectopia. Lateral projection (B) shows a contrast-filled vagina (arrow), separate and posterior to the urinary bladder (arrowhead)

**Figure 3:**
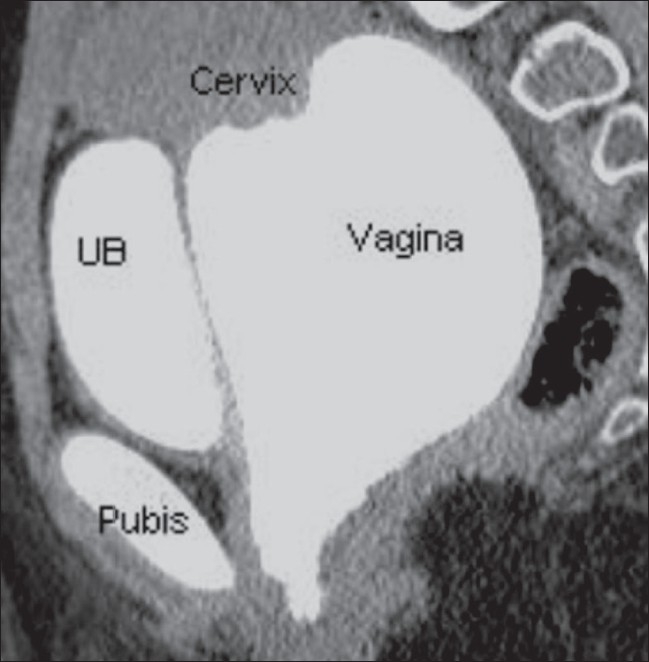
Sagittal reformatted CT scan of the pelvis shows a contrastfilled vagina, separate and posterior to the urinary bladder, without any anomalous connection/extravasation

**Figure 4(A–D):**
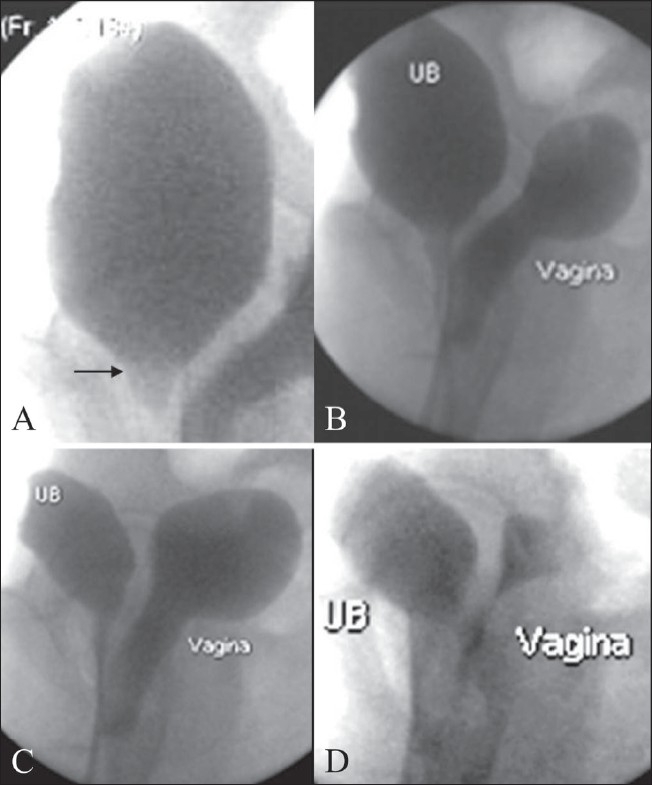
Voiding cystourethrogram. (A) Widened bladder neck is seen (arrow). The early voiding phases (B,C) show gross distension of the vagina due to retrograde filling with progressive bladder (UB) emptying. Late voiding phase (D) shows subtotal evacuation of the vagina and a urinary bladder residue

A 14-year-old adolescent, weighing 45 kg, with a normal menstrual history presented with urinary incontinence since childhood. Clinical examination revealed normal external genitalia. There was continuous dribbling of urine from the vagina. On limited per vaginal examination, the introitus admitted one finger and revealed a ballooned-out vagina. Renal function tests and blood counts were within normal limits. Urine examination revealed numerous pus cells, red blood cells and epithelial cells. There was significant bacteriuria (> 1 00 000 colony-forming units/ml); *Escherichia coli* was the organism isolated. Urodynamic study demonstrated interrupted voiding flow curves. Ultrasonography of the abdomen and pelvis revealed a grossly distended fluid-filled vagina that was suggestive of hydrocolpos [Figures 1A, B]. The uterus, both ovaries and the urinary bladder were normal [Figure 1C]. Postmicturition study showed complete evacuation of the vaginal fluid and postvoid residual urine of 50 ml in the urinary bladder [Figure 1D]. The ureteric jets on both sides were normally seen within the bladder. No obvious reproductive tract abnormalities were seen. An intravenous pyelogram (IVP) did not reveal any ureteral ectopia [Figure 2A]. A separate contrast-filled sac was seen posterior to the urinary bladder in the lateral projection of the pelvis, consistent with a distended vagina [Figure 2B]. Limited CT sections of the pelvis [Figure 3] were obtained in the same sitting as the IVP so as to rule out a vesicovaginal fistula and confirm the absence of ureteral ectopia. Voiding cystourethrography revealed a normal filling phase, without any extravasation. A widened urinary bladder neck was noted [Figure 4A]. The early voiding phase demonstrated progressive gross distention of the vagina due to retrograde filling as the bladder emptied [Figures 4B and C]. The late voiding phase demonstrated progressive complete evacuation of the vagina [Figure 4D]. No vesicoureteral reflux (VUR) was seen bilaterally.

## Discussion

Even though VVR is commonly encountered, it is an uncommon cause of hydrocolpos. Vesicovaginal reflux causes retrograde filling of the vagina during micturition. It can occur in both, the supine and the upright positions.[1] Urinary incontinence, recurrent urinary tract infection (UTI), wetting, vulvovaginitis, irritation of the genitalia, bad smell and vaginal discharge may be the various presentations.[1,3,5] The UTI may be real or due to contamination of urine by the vaginal flora. The condition is common in prepubertal children; however, it may also be seen in postpubertal girls and women.[3] The vaginal distention may be complete, partial or minimal; gross distention is relatively uncommon.[4] The urogenital tract anatomy is usually normal for age.[3,4] A relatively horizontal vagina in the prepubertal age, tightly apposed labia in obese subjects, labia minora adhesions, hypospadiasis and spastic pelvic floor muscles (as seen in patients with cerebral palsy) are the various etiologies proposed for the occurrence of VVR.[1,3,5–7] The diagnosis of VVR is indicated by resolution of the hydrocolpos on a postvoid USG and can be confirmed with a voiding cystourethrogram, which shows gradual distension of the vagina during micturition due to its retrograde filling as the bladder empties. A wide bladder neck, as seen in our patient, a spinning top urethra or low-bladder volumes may be the associated functional voiding disturbances.[1,8,9]

Gross hydrocolpos makes the present case unusual. Absence of hydrometra and a normal menstrual history ruled out an imperforate hymen. The fluid-filled vagina seen posterior to the distended urinary bladder could have been confused with a distended rectum on USG; however, this was ruled out on seeing the cervix suspended at its upper end. Instructions on proper voiding form a key element in the management of VVR.[3]

## References

[1] Snyder EM, Nguyen RA, Young KJ, Coley BD (2007). Vesicovaginal reflux mimicking obstructive hydrocolpos. J Ultrasound Med.

[2] Schaffer RM, Taylor C, Haller JO, Friedman AP, Shih YH (1983). Nonobstructive Hydrocolpos: Sonographic appearance and differential diagnosis. Radiology.

[3] Mattsson S, Gladh G (2003). Urethrovaginal reflux-a common cause of daytime incontinence in girls. Pediatrics.

[4] Kelalis PP, Burke EC, Stickler GB, Hartman GW (1973). Urinary vaginal reflux in children. Pediatrics.

[5] Capraro VJ, Greenberg H (1972). Adhesions of the labia minora: A study of 50 patients. Obstet Gynecol.

[6] Stannard MW, Lebowitz RL (1978). Urography in the child who wets. AJR Am J Roentgenol.

[7] Szabó L, Lombay B, Borbás E, Bajusz (2004). Videourodynamics in the diagnosis of urinary tract abnormalities in a single center. Pediatr Nephrol.

[8] Hausegger KA, Fotter R, Sorantin E, Schmidt P (1991). Urethral morphology and bladder instability. Pediatr Radiol.

[9] Saxton HM, Borzyskowski M, Mundy AR, Vivian GC (1988). Spinning top urethra: not a normal variant. Radiology.

